# Pseudohyperkalemia

**DOI:** 10.4103/0972-5229.40953

**Published:** 2008

**Authors:** F. Kapadia

**Affiliations:** Hinduja Hospital and Research Center, Mumbai, India

Sir,

In their case report titled “Severe hyperkalemia with normal electrocardiogram” “the authors authors report a serum potassium level of 11.3 without electrocardiogram (ECG) changes. The serum potassium is out of proportion to the degree of acidosis and azotemia. They also report a white blood cell count of (WBC) 31.4 and a platelet count of 834. They mention fictitious or pseudohyperkalemia, but do not explore it adequately. One form of pseudohyperkalemia occurs in patients with high WBCs or platelets. This occurs as an *in vitro* effect during clot formation in the test tube with extrusion of potassium from the excess number of WBCs or platelets. The clue to such a situation lies in the very high potassium and a normal ECG. The confirmation is done by measuring the plasma potassium (normal) rather than serum potassium (high). I suspect that this child's hyperkalemia was partly due to this.”

